# Crystal structure of cholest-5-en-3β-yl 3-(2,4-dimeth­oxy-3-methyl­phen­yl)prop-2-enoate

**DOI:** 10.1107/S2056989014028278

**Published:** 2015-01-10

**Authors:** Bernhard Bugenhagen, Ariane Munk, Volkmar Vill, Yosef Al Jasem, Thies Thiemann

**Affiliations:** a Institute of Inorganic Chemistry, University of Hamburg, Hamburg, Germany; b Institute of Organic Chemistry, University of Hamburg, Hamburg, Germany; c Department of Chemical Engineering, United Arab Emirates University, Al Ain, Abu Dhabi; d Department of Chemistry, United Arab Emirates University, Al Ain, Abu Dhabi

**Keywords:** crystal structure, cholesteryl cinnamates, methyl (*E*)-3-(2,4-dimeth­oxy-3-methyl­phen­yl)acrylate, hydrogen bonding, C—H⋯π inter­actions

## Abstract

In the title compound, C_39_H_58_O_4_, the steroid rings *A* and *C* adopt a chair conformation, while ring *B* adopts a half-chair conformation, and ring *D* has an envelope conformation, with the methyl-substituted C atom as the flap. In the crystal, mol­ecules pack within layers parallel to (100), with their long axis parallel to the [101] direction. Adjacent layers are linked *via* C—H⋯O hydrogen bonds and C—H⋯π inter­actions, forming a three-dimensional framework.

## Related literature   

For the preparation of the title compound, see: Thiemann *et al.* (2011 ▸). For applications of cholesteryl cinnamates, see: Vora (1976 ▸); Kutulya *et al.* (1983 ▸); Tanaka *et al.* (1981 ▸); Dong *et al.* (2010 ▸). For the crystal structure of a similar compound, see: Bugenhagen *et al.* (2012 ▸).
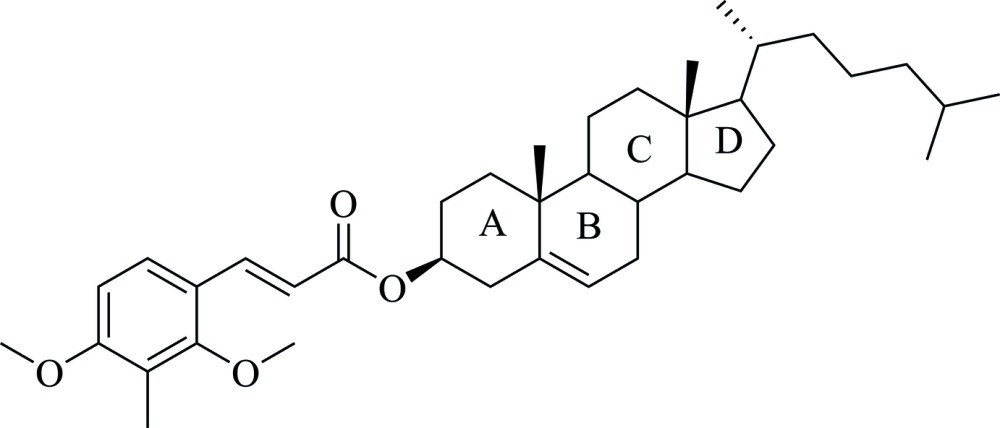


## Experimental   

### Crystal data  

C_39_H_58_O_4_
*M* *_r_* = 590.85
Orthorhombic, 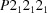

*a* = 9.4626 (6) Å
*b* = 12.2687 (8) Å
*c* = 29.6074 (18) Å
*V* = 3437.2 (4) Å^3^
*Z* = 4
Mo *K*α radiation
μ = 0.07 mm^−1^
*T* = 100 K
0.25 × 0.07 × 0.07 mm

### Data collection  

Bruker SMART APEX CCD area-detector diffractometer
Absorption correction: multi-scan (*SADABS*; Bruker, 2009 ▸) *T*_min_ = 0.702, *T*_max_ = 0.746
33550 measured reflections
4399 independent reflections
3563 reflections with *I* > 2σ(*I*)
*R*_int_ = 0.052

### Refinement  

*R*[*F* ^2^ > 2σ(*F* ^2^)] = 0.046
*wR*(*F* ^2^) = 0.112
*S* = 1.03
4399 reflections
396 parameters
H-atom parameters constrained
Δρ_max_ = 0.56 e Å^−3^
Δρ_min_ = −0.20 e Å^−3^

### 

Data collection: *APEX2* (Bruker, 2009 ▸); cell refinement: *SAINT* (Bruker, 2009 ▸); data reduction: *SAINT*; program(s) used to solve structure: *SHELXS97* (Sheldrick, 2008 ▸); program(s) used to refine structure: *SHELXL97* (Sheldrick, 2008 ▸); molecular graphics: *PLATON* (Spek, 2009 ▸) and *Mercury* (Macrae *et al.*, 2008 ▸); software used to prepare material for publication: *OLEX2* (Dolomanov *et al.*, 2009 ▸).

## Supplementary Material

Crystal structure: contains datablock(s) I. DOI: 10.1107/S2056989014028278/su5049sup1.cif

Structure factors: contains datablock(s) I. DOI: 10.1107/S2056989014028278/su5049Isup2.hkl

**Fig. 1.**
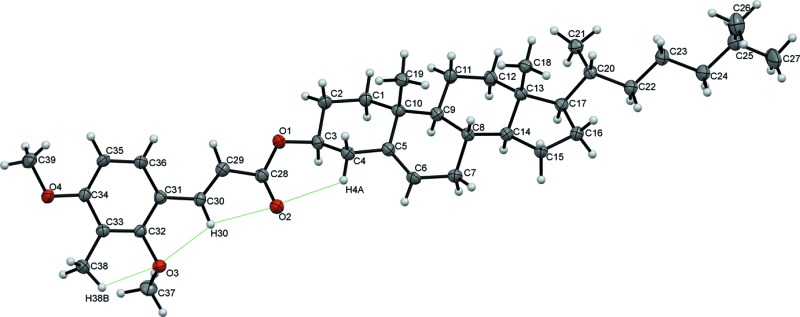
A view of molecular structure of the title molecule, with atom labelling. Displacement ellipsoids are shown at the 50% probability level. The short intramolecular C-H···O contacts are shown as green dashed lines (see Table 1 for details).

. DOI: 10.1107/S2056989014028278/su5049fig1.tif
A view of mol­ecular structure of the title mol­ecule, with atom labelling. Displacement ellipsoids are shown at the 50% probability level. The short intra­molecular C-H⋯O contacts are shown as green dashed lines (see Table 1 for details).

**Fig. 2.**
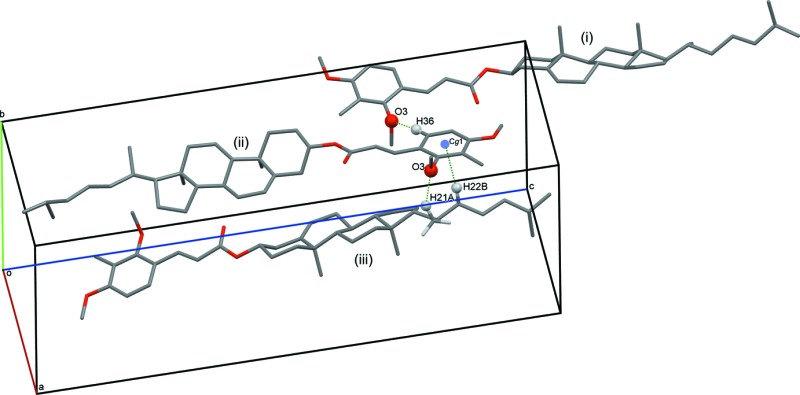
Intermolecular C—H···O and C—H···π(Cg1) contacts between molecules of the title compound (see Table 1 for details; symmetry codes: (i) - x + 1, y + 1/2, - z +3/2; (ii) x, y, z; (iii) x + 1/2, - y + 3/2, - z + 1).

Click here for additional data file.Cg x y z x y z x y z . DOI: 10.1107/S2056989014028278/su5049fig2.tif
Inter­molecular C—H⋯O and C—H⋯π(*Cg*1) contacts between mol­ecules of the title compound (see Table 1 for details; symmetry codes: (i) − *x* + 1, *y* + 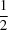, − *z* + 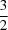; (ii) *x*, *y*, *z*; (iii) *x* + 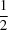, − *y* + 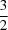, − *z* + 1).

**Fig. 3.**
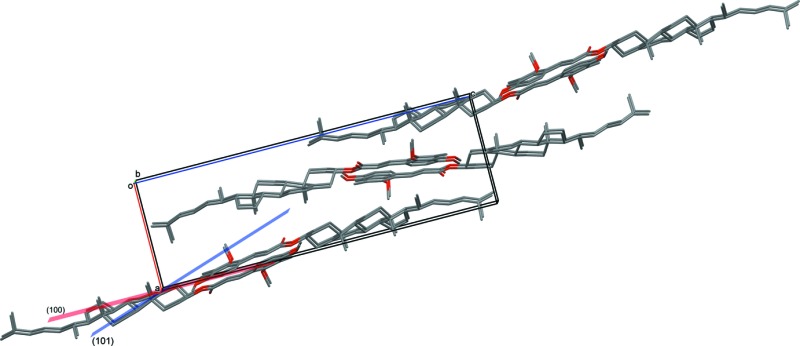
A view of adjacent molecules lying in layers (three layers in this figure) parallel to (100), showing their long molecular axis which is parallel to the [101] direction.

. DOI: 10.1107/S2056989014028278/su5049fig3.tif
A view of adjacent mol­ecules lying in layers (three layers in this figure) parallel to (100), showing their long mol­ecular axis which is parallel to the [101] direction.

CCDC reference: 884045

Additional supporting information:  crystallographic information; 3D view; checkCIF report

## Figures and Tables

**Table 1:** Hydrogen-bond geometry (, ) *Cg*1 is the centroid of the C31C36 ring.

*D*H*A*	*D*H	H*A*	*D* *A*	*D*H*A*
C21H21*A*O3^i^	0.98	2.57	3.399(3)	143
C36H36O3^ii^	0.95	2.51	3.431(3)	164
C22H22*B* *Cg*1^i^	0.99	2.77	3.756(3)	173

Symmetry codes: (i) 
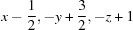
; (ii) 
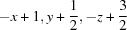
.

## supplementary crystallographic information

### S1. Structural commentary

Cholesteryl cinnamates exhibit chiral mesogenic phases. The influence of the substituents of the cinnamyl unit in these compounds on their phase transition behaviour (Vora, 1976; Kutulya *et al.*, 1983) remains of inter­est (Bugenhagen *et al.*, 2012). Also, their crystal packing at room temperature which can give the possibility to photodimerize the substances in the crystal (Tanaka *et al.*, 1981; Dong *et al.*, 2010) is continued to be studied. For the title compound, the authors have observed the following phase transformation sequence: Cr 162.2 Ch 229.9 I, where the numbers denote temperature of the phase transition in °C.

There are short intra­molecular C—H···O contacts present in the methyl (E)-3-(2,4-di­meth­oxy-3-methyl­phenyl)­acrylate moiety of the title compound (Fig. 1 and Table 1).

The conformational analysis of rings A, B, C and D was carried out. It was found that rings A and C adopt a chair conformation, while ring B adopts a half-chair conformation, and ring D adopts an envelope conformation with the methyl substituted C atom as the flap.

In the crystal, molecules are arranged in separate layers parallel to (1 0 0). Within each layer, translation related molecules form columns extend along [1 0 1] with their long molecular axis collinear with this direction (Figure 3). Molecules in the neighbouring columns exhibit head to tail arrangement with C—H···O inter­actions occurring between the 2-meth­oxy group (O3) of one cinnamate unit with the C6—H (H36) of the other molecule's cinnamate unit (Table 1). The neighboring layers are packed in such a manner that there are two close contact, (C21—H21A···O3) and (C22—H22B···π) between molecules in a head-to-tail arrangement, with a dihedral angle between the steroidal mean planes of these contacting molecules of 46.7 (2)° (Fig. 2 and Table 1). These inter­actions lead to the formation of a three-dimensional framework.

### S2. Synthesis and crystallization

To a solution of tri­phenyl­phosphine (582 mg, 2.2 mmol) in CH_2_Cl_2_ (7.5 mL) is added bromo­tri­chloro­methane (900 mg, 4.5 mmol), and the resulting solution is stirred for 20 min. at rt. Thereafter, 3-(2',4'-di­meth­oxy-3-'methyl­phenyl)­prop-2-enoic acid (2,4-di­meth­oxy-3-methyl­cinnamic acid, 444 mg, 2.0 mmol) is added, and the solution is heated at 323 K for 15 min. Cholest-5-en-3β-ol (cholesterol, 386 mg, 1.0 mmol) is added, and after 20 min. Et_3_N (200 mg, 2.0 mmol) is added dropwise with the help of a syringe. The reaction mixture is stirred at 318 K for 12h. Then, it is cooled, poured into water (30 mL) and extracted with CH_2_Cl_2_ (3 × 15 mL). The organic phase is washed with 15 w% aq. NaOH (15 mL) and subsequently with aq. HCl (1 mL conc. HCl in 7 mL of H_2_O), dried over anhydrous MgSO_4_, and evaporated *in vacuo*. Column chromatography of the residue on silica gel (eluent M*t*BE/hexane/CHCl_3_ 1:3:1) gives the target compound (413 mg, 70%) as colorless needles; *δ*_H_ (400 MHz, CDCl_3_) 0.68 (3H, s, CH_3_), 0.86 (3H, d, ^3^*J* = 6.8 Hz, CH_3_), 0.87 (3H, d, ^3^*J* = 6.8 Hz, CH_3_), 0.92 (3H, d, ^3^*J* = 6.4 Hz, CH_3_), 0.98 – 2.17 (26H, m), 1.05 (3H, s, CH_3_), 2.41 (2H, m), 3.73 (3H, s, OCH_3_), 3.85 (3H, s, OCH_3_), 4.73 (1H, m), 5.41 (1H, m), 6.38 (1H, d, ^3^*J* = 16.0 Hz), 6.66 (1H, d, ^3^*J* = 8.7 Hz), 7.41 (1H, d, ^3^*J* = 8.7 Hz), 7.89 (1H, d, ^3^*J* = 16.0 Hz); *δ*_C_ (100.5 MHz, CDCl_3_) 8.9, 11.9, 18.7, 19.4, 21.0, 22.6, 22.8, 23.8, 24.3, 27.9, 28.0, 28.2, 31.9, 35.8, 36.2, 36.6, 37.0, 38.3, 39.5, 39.7, 42.3, 50.0, 55.7, 56.1, 56.7, 61.5, 73.8, 106.5, 117.0, 120.2, 120.7, 122.6, 126.0, 139.8, 139.9, 158.9, 160.5, 167.0.

### S3. Refinement

All H atoms were placed in calculated positions with C—H distances of 0.95 - 1.00 Å and refined as riding with *U*_iso_(H) = 1.5*U*_eq_(C) for methyl H atoms and = 1.2U_eq_(C) for other H-atoms. In the final cycles of refinement, in the absence of significant anomalous scattering effects, the Friedel pairs were merged and Δf ' set to zero.

### Crystal data

**Table:**

C_39_H_58_O_4_	*D*_x_ = 1.142 Mg m^−^^3^
*M**_r_* = 590.85	Melting point: 503 K
Orthorhombic, *P*2_1_2_1_2_1_	Mo *K*α radiation, λ = 0.71073 Å
*a* = 9.4626 (6) Å	Cell parameters from 4605 reflections
*b* = 12.2687 (8) Å	θ = 2.3–20.4°
*c* = 29.6074 (18) Å	µ = 0.07 mm^−^^1^
*V* = 3437.2 (4) Å^3^	*T* = 100 K
*Z* = 4	Needle, colourless
*F*(000) = 1296	0.25 × 0.07 × 0.07 mm

### Data collection

**Table:**

Bruker SMART APEX CCD area-detector diffractometer	4399 independent reflections
Radiation source: micro-focus	3563 reflections with *I* > 2σ(*I*)
Multi-layer monochromator	*R*_int_ = 0.052
Detector resolution: 8 pixels mm^-1^	θ_max_ = 27.5°, θ_min_ = 2.2°
ω and φ scans	*h* = −12→12
Absorption correction: multi-scan (*SADABS*; Bruker, 2009)	*k* = −15→15
*T*_min_ = 0.702, *T*_max_ = 0.746	*l* = −38→38
33550 measured reflections	

### Refinement

**Table:**

Refinement on *F*^2^	Primary atom site location: structure-invariant direct methods
Least-squares matrix: full	Secondary atom site location: difference Fourier map
*R*[*F*^2^ > 2σ(*F*^2^)] = 0.046	Hydrogen site location: inferred from neighbouring sites
*wR*(*F*^2^) = 0.112	H-atom parameters constrained
*S* = 1.03	*w* = 1/[σ^2^(*F*_o_^2^) + (0.0454*P*)^2^ + 1.4494*P*] where *P* = (*F*_o_^2^ + 2*F*_c_^2^)/3
4399 reflections	(Δ/σ)_max_ = 0.001
396 parameters	Δρ_max_ = 0.56 e Å^−^^3^
0 restraints	Δρ_min_ = −0.20 e Å^−^^3^

### Special details

**Table:**

Geometry. All e.s.d.'s (except the e.s.d. in the dihedral angle between two l.s. planes) are estimated using the full covariance matrix. The cell e.s.d.'s are taken into account individually in the estimation of e.s.d.'s in distances, angles and torsion angles; correlations between e.s.d.'s in cell parameters are only used when they are defined by crystal symmetry. An approximate (isotropic) treatment of cell e.s.d.'s is used for estimating e.s.d.'s involving l.s. planes.
Refinement. Refinement of *F*^2^ against ALL reflections. The weighted *R*-factor *wR* and goodness of fit *S* are based on *F*^2^, conventional *R*-factors *R* are based on *F*, with *F* set to zero for negative *F*^2^. The threshold expression of *F*^2^ > σ(*F*^2^) is used only for calculating *R*-factors(gt) *etc*. and is not relevant to the choice of reflections for refinement. *R*-factors based on *F*^2^ are statistically about twice as large as those based on *F*, and *R*-factors based on ALL data will be even larger.

### Fractional atomic coordinates and isotropic or equivalent isotropic displacement parameters (Å^2^)

**Table:**

	*x*	*y*	*z*	*U*_iso_*/*U*_eq_	
C1	0.3236 (3)	0.9302 (2)	0.48990 (8)	0.0265 (6)	
C10	0.4360 (3)	0.8630 (2)	0.46390 (8)	0.0231 (6)	
C11	0.3496 (3)	0.9329 (2)	0.38602 (8)	0.0275 (6)	
C12	0.3055 (3)	0.9041 (2)	0.33754 (8)	0.0278 (6)	
C13	0.4131 (3)	0.8268 (2)	0.31576 (8)	0.0228 (6)	
C14	0.4170 (3)	0.7240 (2)	0.34577 (8)	0.0242 (6)	
C15	0.5026 (3)	0.6419 (2)	0.31835 (8)	0.0264 (6)	
C16	0.4683 (3)	0.6701 (2)	0.26855 (8)	0.0267 (6)	
C17	0.3738 (3)	0.7732 (2)	0.26950 (8)	0.0239 (6)	
C18	0.5575 (3)	0.8817 (2)	0.31194 (9)	0.0260 (6)	
C19	0.5755 (3)	0.9279 (2)	0.45997 (9)	0.0286 (6)	
C2	0.3567 (3)	0.9469 (2)	0.54015 (9)	0.0284 (6)	
C20	0.3855 (3)	0.8421 (2)	0.22638 (8)	0.0235 (6)	
C21	0.2799 (3)	0.9367 (2)	0.22650 (9)	0.0283 (6)	
C22	0.3674 (3)	0.7725 (2)	0.18347 (8)	0.0280 (6)	
C23	0.4106 (3)	0.8306 (2)	0.14017 (8)	0.0319 (7)	
C24	0.4104 (4)	0.7584 (2)	0.09850 (8)	0.0345 (7)	
C25	0.4636 (4)	0.8142 (3)	0.05567 (9)	0.0349 (7)	
C26	0.6193 (4)	0.8428 (3)	0.05855 (11)	0.0492 (9)	
C27	0.4329 (6)	0.7462 (4)	0.01422 (10)	0.0776 (16)	
C28	0.4001 (3)	0.7813 (2)	0.64075 (9)	0.0253 (6)	
C29	0.4332 (3)	0.8217 (2)	0.68646 (8)	0.0256 (6)	
C3	0.3759 (3)	0.8379 (2)	0.56332 (8)	0.0256 (6)	
C30	0.4294 (3)	0.7554 (2)	0.72230 (8)	0.0224 (5)	
C31	0.4576 (3)	0.7846 (2)	0.76936 (8)	0.0225 (5)	
C32	0.4539 (3)	0.7051 (2)	0.80346 (8)	0.0215 (5)	
C33	0.4829 (3)	0.7288 (2)	0.84855 (8)	0.0231 (6)	
C34	0.5126 (3)	0.8372 (2)	0.85963 (8)	0.0239 (6)	
C35	0.5165 (3)	0.9187 (2)	0.82683 (8)	0.0252 (6)	
C36	0.4898 (3)	0.8915 (2)	0.78239 (8)	0.0257 (6)	
C37	0.2873 (3)	0.5625 (3)	0.80063 (10)	0.0344 (7)	
C38	0.4917 (3)	0.6416 (2)	0.88433 (8)	0.0287 (6)	
C39	0.5834 (4)	0.9622 (2)	0.91719 (10)	0.0398 (8)	
C4	0.4913 (3)	0.7723 (2)	0.54083 (8)	0.0263 (6)	
C5	0.4638 (3)	0.7582 (2)	0.49066 (8)	0.0239 (6)	
C6	0.4669 (3)	0.6604 (2)	0.47162 (8)	0.0259 (6)	
C7	0.4521 (3)	0.6396 (2)	0.42197 (8)	0.0290 (6)	
C8	0.4648 (3)	0.7443 (2)	0.39412 (8)	0.0233 (6)	
C9	0.3748 (3)	0.8333 (2)	0.41646 (8)	0.0238 (6)	
H11A	0.2749	0.9788	0.3998	0.033*	
H11B	0.4373	0.9768	0.3849	0.033*	
H12A	0.2113	0.8690	0.3379	0.033*	
H12B	0.2984	0.9716	0.3194	0.033*	
H14	0.3181	0.6954	0.3474	0.029*	
H15A	0.4737	0.5663	0.3256	0.032*	
H15B	0.6050	0.6499	0.3245	0.032*	
H16A	0.5563	0.6846	0.2515	0.032*	
H16B	0.4178	0.6089	0.2539	0.032*	
H17	0.2736	0.7480	0.2718	0.029*	
H18A	0.5943	0.8968	0.3422	0.039*	
H18B	0.5479	0.9503	0.2952	0.039*	
H18C	0.6228	0.8334	0.2959	0.039*	
H19A	0.6409	0.8888	0.4400	0.043*	
H19B	0.6181	0.9356	0.4900	0.043*	
H19C	0.5560	1.0002	0.4474	0.043*	
H1A	0.3144	1.0025	0.4754	0.032*	
H1B	0.2312	0.8929	0.4872	0.032*	
H20	0.4826	0.8742	0.2256	0.028*	
H21A	0.1861	0.9095	0.2349	0.042*	
H21B	0.2756	0.9693	0.1963	0.042*	
H21C	0.3103	0.9919	0.2484	0.042*	
H22A	0.4247	0.7054	0.1866	0.034*	
H22B	0.2671	0.7503	0.1809	0.034*	
H23A	0.3453	0.8924	0.1350	0.038*	
H23B	0.5066	0.8611	0.1443	0.038*	
H24A	0.3128	0.7323	0.0932	0.041*	
H24B	0.4702	0.6938	0.1045	0.041*	
H25	0.4103	0.8841	0.0524	0.042*	
H26A	0.6355	0.8900	0.0847	0.074*	
H26B	0.6480	0.8809	0.0310	0.074*	
H26C	0.6748	0.7758	0.0618	0.074*	
H27A	0.4889	0.6790	0.0153	0.116*	
H27B	0.4578	0.7876	−0.0129	0.116*	
H27C	0.3321	0.7278	0.0134	0.116*	
H29	0.4576	0.8961	0.6905	0.031*	
H2A	0.4440	0.9907	0.5433	0.034*	
H2B	0.2784	0.9873	0.5547	0.034*	
H3	0.2851	0.7962	0.5622	0.031*	
H30	0.4059	0.6813	0.7166	0.027*	
H35	0.5372	0.9919	0.8349	0.030*	
H36	0.4935	0.9469	0.7600	0.031*	
H37A	0.2203	0.6075	0.7836	0.052*	
H37B	0.2683	0.5697	0.8330	0.052*	
H37C	0.2767	0.4860	0.7917	0.052*	
H38A	0.5758	0.6538	0.9030	0.043*	
H38B	0.4981	0.5699	0.8699	0.043*	
H38C	0.4071	0.6445	0.9034	0.043*	
H39A	0.6663	0.9831	0.8994	0.060*	
H39B	0.6076	0.9631	0.9494	0.060*	
H39C	0.5063	1.0137	0.9115	0.060*	
H4A	0.4973	0.6997	0.5553	0.032*	
H4B	0.5830	0.8096	0.5452	0.032*	
H6	0.4794	0.5991	0.4908	0.031*	
H7A	0.3591	0.6056	0.4160	0.035*	
H7B	0.5262	0.5877	0.4123	0.035*	
H8	0.5659	0.7682	0.3939	0.028*	
H9	0.2799	0.8001	0.4220	0.029*	
O1	0.4114 (2)	0.86254 (15)	0.61032 (6)	0.0278 (4)	
O2	0.3647 (2)	0.68939 (15)	0.63125 (6)	0.0299 (5)	
O3	0.4279 (2)	0.59773 (14)	0.79131 (6)	0.0237 (4)	
O4	0.5397 (2)	0.85517 (14)	0.90460 (6)	0.0304 (5)	

### Atomic displacement parameters (Å^2^)

**Table:**

	*U*^11^	*U*^22^	*U*^33^	*U*^12^	*U*^13^	*U*^23^
C1	0.0330 (16)	0.0255 (14)	0.0212 (13)	0.0032 (12)	0.0000 (11)	−0.0011 (11)
C10	0.0248 (14)	0.0223 (13)	0.0222 (12)	0.0015 (11)	−0.0009 (11)	0.0022 (10)
C11	0.0340 (16)	0.0267 (14)	0.0219 (13)	0.0069 (13)	0.0003 (11)	−0.0004 (11)
C12	0.0317 (16)	0.0291 (15)	0.0226 (13)	0.0047 (13)	−0.0003 (12)	0.0012 (12)
C13	0.0242 (13)	0.0231 (13)	0.0211 (12)	0.0016 (11)	0.0006 (11)	0.0012 (11)
C14	0.0292 (14)	0.0220 (13)	0.0213 (12)	−0.0011 (12)	0.0021 (11)	0.0002 (10)
C15	0.0348 (16)	0.0225 (13)	0.0218 (12)	0.0009 (12)	0.0019 (11)	−0.0006 (11)
C16	0.0332 (15)	0.0240 (13)	0.0228 (12)	0.0006 (12)	0.0024 (12)	−0.0017 (11)
C17	0.0245 (14)	0.0238 (13)	0.0234 (12)	−0.0008 (12)	0.0003 (11)	−0.0013 (11)
C18	0.0305 (15)	0.0230 (13)	0.0246 (13)	−0.0021 (12)	−0.0027 (12)	0.0028 (11)
C19	0.0339 (16)	0.0266 (14)	0.0254 (13)	−0.0057 (13)	−0.0012 (12)	0.0020 (11)
C2	0.0391 (16)	0.0229 (14)	0.0233 (13)	0.0057 (13)	0.0011 (12)	0.0006 (11)
C20	0.0217 (13)	0.0269 (14)	0.0219 (12)	−0.0029 (11)	−0.0013 (11)	−0.0002 (11)
C21	0.0301 (15)	0.0274 (15)	0.0273 (13)	−0.0013 (12)	−0.0038 (12)	0.0016 (12)
C22	0.0324 (15)	0.0282 (14)	0.0233 (13)	−0.0012 (13)	−0.0023 (12)	−0.0020 (12)
C23	0.0379 (17)	0.0343 (15)	0.0236 (13)	−0.0024 (14)	−0.0013 (12)	−0.0033 (12)
C24	0.0469 (19)	0.0323 (16)	0.0243 (13)	−0.0058 (14)	−0.0044 (13)	−0.0014 (12)
C25	0.0486 (19)	0.0333 (16)	0.0227 (13)	−0.0015 (15)	−0.0017 (14)	0.0005 (12)
C26	0.048 (2)	0.066 (2)	0.0335 (16)	0.0039 (19)	0.0100 (15)	−0.0044 (17)
C27	0.144 (5)	0.067 (3)	0.0216 (16)	−0.045 (3)	−0.004 (2)	−0.0024 (18)
C28	0.0277 (15)	0.0250 (14)	0.0231 (13)	0.0035 (12)	0.0015 (11)	0.0016 (11)
C29	0.0283 (14)	0.0237 (13)	0.0249 (13)	0.0001 (12)	0.0012 (11)	−0.0014 (11)
C3	0.0334 (15)	0.0259 (14)	0.0174 (12)	−0.0022 (12)	−0.0031 (11)	0.0014 (11)
C30	0.0232 (13)	0.0226 (13)	0.0215 (12)	0.0015 (11)	0.0028 (11)	−0.0017 (11)
C31	0.0220 (13)	0.0233 (13)	0.0222 (12)	0.0018 (11)	0.0018 (11)	−0.0003 (10)
C32	0.0223 (13)	0.0192 (13)	0.0231 (12)	0.0011 (11)	0.0013 (11)	−0.0020 (10)
C33	0.0253 (14)	0.0214 (13)	0.0227 (12)	0.0016 (12)	0.0003 (11)	0.0005 (10)
C34	0.0283 (14)	0.0240 (13)	0.0194 (11)	0.0028 (12)	0.0017 (11)	−0.0032 (10)
C35	0.0344 (15)	0.0168 (12)	0.0245 (12)	0.0018 (12)	−0.0009 (12)	−0.0026 (10)
C36	0.0331 (16)	0.0211 (13)	0.0230 (12)	0.0024 (12)	0.0022 (12)	0.0018 (11)
C37	0.0279 (16)	0.0366 (17)	0.0387 (16)	−0.0086 (14)	0.0036 (13)	−0.0003 (14)
C38	0.0382 (16)	0.0252 (13)	0.0227 (12)	−0.0004 (13)	−0.0016 (12)	0.0026 (11)
C39	0.066 (2)	0.0264 (15)	0.0270 (14)	−0.0051 (16)	−0.0106 (15)	−0.0039 (12)
C4	0.0308 (15)	0.0248 (13)	0.0233 (12)	0.0004 (12)	−0.0009 (12)	0.0027 (11)
C5	0.0246 (14)	0.0248 (13)	0.0223 (12)	0.0007 (12)	0.0013 (11)	0.0028 (11)
C6	0.0325 (15)	0.0230 (13)	0.0222 (12)	0.0024 (12)	0.0006 (11)	0.0062 (11)
C7	0.0394 (17)	0.0219 (14)	0.0257 (13)	0.0007 (13)	0.0032 (12)	0.0016 (11)
C8	0.0283 (14)	0.0222 (13)	0.0194 (11)	−0.0013 (12)	0.0004 (11)	0.0021 (10)
C9	0.0262 (14)	0.0239 (13)	0.0211 (12)	−0.0010 (12)	0.0011 (11)	0.0011 (11)
O1	0.0404 (12)	0.0245 (10)	0.0186 (8)	−0.0014 (9)	−0.0025 (8)	0.0015 (7)
O2	0.0406 (12)	0.0248 (10)	0.0244 (9)	−0.0029 (9)	−0.0005 (9)	0.0003 (8)
O3	0.0261 (10)	0.0201 (9)	0.0250 (9)	−0.0021 (8)	0.0016 (8)	−0.0009 (7)
O4	0.0490 (13)	0.0220 (9)	0.0200 (9)	−0.0010 (10)	−0.0042 (9)	−0.0020 (7)

### Geometric parameters (Å, º)

**Table:**

O1—C3	1.463 (3)	C32—O3	1.388 (3)
O1—C28	1.348 (3)	C10—C1	1.550 (4)
O2—C28	1.210 (3)	C10—C5	1.533 (4)
C3—H3	1.0000	C10—C19	1.546 (4)
C3—C4	1.511 (4)	C17—H17	1.0000
C3—C2	1.514 (4)	C1—H1A	0.9900
C30—H30	0.9500	C1—H1B	0.9900
C30—C29	1.338 (3)	C1—C2	1.534 (3)
C30—C31	1.463 (3)	C5—C4	1.518 (3)
C34—C33	1.399 (4)	C21—H21A	0.9800
C34—C35	1.394 (4)	C21—H21B	0.9800
C34—O4	1.374 (3)	C21—H21C	0.9800
C8—H8	1.0000	C12—H12A	0.9900
C8—C14	1.522 (3)	C12—H12B	0.9900
C8—C7	1.531 (3)	C12—C11	1.536 (4)
C8—C9	1.535 (4)	C22—H22A	0.9900
C14—H14	1.0000	C22—H22B	0.9900
C14—C15	1.526 (4)	C22—C23	1.523 (4)
C14—C13	1.544 (4)	C4—H4A	0.9900
C28—C29	1.475 (4)	C4—H4B	0.9900
C29—H29	0.9500	C2—H2A	0.9900
C15—H15A	0.9900	C2—H2B	0.9900
C15—H15B	0.9900	C24—H24A	0.9900
C15—C16	1.549 (3)	C24—H24B	0.9900
C7—H7A	0.9900	C24—C23	1.519 (4)
C7—H7B	0.9900	C11—H11A	0.9900
C7—C6	1.499 (3)	C11—H11B	0.9900
C6—H6	0.9500	C23—H23A	0.9900
C6—C5	1.326 (4)	C23—H23B	0.9900
C31—C36	1.401 (4)	C26—H26A	0.9800
C31—C32	1.404 (3)	C26—H26B	0.9800
C33—C32	1.394 (3)	C26—H26C	0.9800
C33—C38	1.508 (4)	O4—C39	1.426 (3)
C20—H20	1.0000	O3—C37	1.426 (3)
C20—C17	1.535 (3)	C18—H18A	0.9800
C20—C21	1.532 (4)	C18—H18B	0.9800
C20—C22	1.540 (3)	C18—H18C	0.9800
C13—C17	1.564 (3)	C19—H19A	0.9800
C13—C12	1.534 (4)	C19—H19B	0.9800
C13—C18	1.527 (4)	C19—H19C	0.9800
C35—H35	0.9500	C38—H38A	0.9800
C35—C36	1.381 (3)	C38—H38B	0.9800
C16—H16A	0.9900	C38—H38C	0.9800
C16—H16B	0.9900	C37—H37A	0.9800
C16—C17	1.549 (4)	C37—H37B	0.9800
C36—H36	0.9500	C37—H37C	0.9800
C25—H25	1.0000	C39—H39A	0.9800
C25—C24	1.526 (4)	C39—H39B	0.9800
C25—C26	1.516 (5)	C39—H39C	0.9800
C25—C27	1.512 (4)	C27—H27A	0.9800
C9—H9	1.0000	C27—H27B	0.9800
C9—C10	1.562 (3)	C27—H27C	0.9800
C9—C11	1.537 (3)		
			
C28—O1—C3	117.7 (2)	C16—C17—H17	107.2
O1—C3—H3	109.5	C10—C1—H1A	108.7
O1—C3—C4	111.3 (2)	C10—C1—H1B	108.7
O1—C3—C2	106.0 (2)	H1A—C1—H1B	107.6
C4—C3—H3	109.5	C2—C1—C10	114.4 (2)
C4—C3—C2	110.9 (2)	C2—C1—H1A	108.7
C2—C3—H3	109.5	C2—C1—H1B	108.7
C29—C30—H30	116.5	C6—C5—C10	122.9 (2)
C29—C30—C31	127.0 (2)	C6—C5—C4	121.0 (2)
C31—C30—H30	116.5	C4—C5—C10	116.1 (2)
C35—C34—C33	121.6 (2)	C20—C21—H21A	109.5
O4—C34—C33	114.7 (2)	C20—C21—H21B	109.5
O4—C34—C35	123.7 (2)	C20—C21—H21C	109.5
C14—C8—H8	109.0	H21A—C21—H21B	109.5
C14—C8—C7	110.2 (2)	H21A—C21—H21C	109.5
C14—C8—C9	110.9 (2)	H21B—C21—H21C	109.5
C7—C8—H8	109.0	C13—C12—H12A	109.5
C7—C8—C9	108.8 (2)	C13—C12—H12B	109.5
C9—C8—H8	109.0	C13—C12—C11	110.8 (2)
C8—C14—H14	106.9	H12A—C12—H12B	108.1
C8—C14—C15	116.8 (2)	C11—C12—H12A	109.5
C8—C14—C13	114.5 (2)	C11—C12—H12B	109.5
C15—C14—H14	106.9	C20—C22—H22A	108.8
C15—C14—C13	104.2 (2)	C20—C22—H22B	108.8
C13—C14—H14	106.9	H22A—C22—H22B	107.7
O1—C28—C29	110.4 (2)	C23—C22—C20	113.9 (2)
O2—C28—O1	123.8 (2)	C23—C22—H22A	108.8
O2—C28—C29	125.8 (2)	C23—C22—H22B	108.8
C30—C29—C28	121.2 (2)	C3—C4—C5	111.6 (2)
C30—C29—H29	119.4	C3—C4—H4A	109.3
C28—C29—H29	119.4	C3—C4—H4B	109.3
C14—C15—H15A	110.9	C5—C4—H4A	109.3
C14—C15—H15B	110.9	C5—C4—H4B	109.3
C14—C15—C16	104.3 (2)	H4A—C4—H4B	108.0
H15A—C15—H15B	108.9	C3—C2—C1	110.3 (2)
C16—C15—H15A	110.9	C3—C2—H2A	109.6
C16—C15—H15B	110.9	C3—C2—H2B	109.6
C8—C7—H7A	109.2	C1—C2—H2A	109.6
C8—C7—H7B	109.2	C1—C2—H2B	109.6
H7A—C7—H7B	107.9	H2A—C2—H2B	108.1
C6—C7—C8	112.2 (2)	C25—C24—H24A	108.7
C6—C7—H7A	109.2	C25—C24—H24B	108.7
C6—C7—H7B	109.2	H24A—C24—H24B	107.6
C7—C6—H6	117.7	C23—C24—C25	114.4 (2)
C5—C6—C7	124.7 (2)	C23—C24—H24A	108.7
C5—C6—H6	117.7	C23—C24—H24B	108.7
C36—C31—C30	122.1 (2)	C9—C11—H11A	108.8
C36—C31—C32	117.2 (2)	C9—C11—H11B	108.8
C32—C31—C30	120.7 (2)	C12—C11—C9	114.0 (2)
C34—C33—C38	119.9 (2)	C12—C11—H11A	108.8
C32—C33—C34	117.5 (2)	C12—C11—H11B	108.8
C32—C33—C38	122.4 (2)	H11A—C11—H11B	107.6
C17—C20—H20	107.6	C22—C23—H23A	108.7
C17—C20—C22	111.9 (2)	C22—C23—H23B	108.7
C21—C20—H20	107.6	C24—C23—C22	114.2 (2)
C21—C20—C17	111.6 (2)	C24—C23—H23A	108.7
C21—C20—C22	110.5 (2)	C24—C23—H23B	108.7
C22—C20—H20	107.6	H23A—C23—H23B	107.6
C14—C13—C17	99.5 (2)	C25—C26—H26A	109.5
C12—C13—C14	106.2 (2)	C25—C26—H26B	109.5
C12—C13—C17	118.0 (2)	C25—C26—H26C	109.5
C18—C13—C14	112.5 (2)	H26A—C26—H26B	109.5
C18—C13—C17	109.5 (2)	H26A—C26—H26C	109.5
C18—C13—C12	110.6 (2)	H26B—C26—H26C	109.5
C34—C35—H35	120.5	C34—O4—C39	117.1 (2)
C36—C35—C34	119.1 (2)	C32—O3—C37	113.8 (2)
C36—C35—H35	120.5	C13—C18—H18A	109.5
C15—C16—H16A	110.4	C13—C18—H18B	109.5
C15—C16—H16B	110.4	C13—C18—H18C	109.5
H16A—C16—H16B	108.6	H18A—C18—H18B	109.5
C17—C16—C15	106.7 (2)	H18A—C18—H18C	109.5
C17—C16—H16A	110.4	H18B—C18—H18C	109.5
C17—C16—H16B	110.4	C10—C19—H19A	109.5
C31—C36—H36	119.1	C10—C19—H19B	109.5
C35—C36—C31	121.9 (2)	C10—C19—H19C	109.5
C35—C36—H36	119.1	H19A—C19—H19B	109.5
C24—C25—H25	107.3	H19A—C19—H19C	109.5
C26—C25—H25	107.3	H19B—C19—H19C	109.5
C26—C25—C24	112.2 (3)	C33—C38—H38A	109.5
C27—C25—H25	107.3	C33—C38—H38B	109.5
C27—C25—C24	111.3 (3)	C33—C38—H38C	109.5
C27—C25—C26	111.1 (3)	H38A—C38—H38B	109.5
C8—C9—H9	106.3	H38A—C38—H38C	109.5
C8—C9—C10	110.4 (2)	H38B—C38—H38C	109.5
C8—C9—C11	113.6 (2)	O3—C37—H37A	109.5
C10—C9—H9	106.3	O3—C37—H37B	109.5
C11—C9—H9	106.3	O3—C37—H37C	109.5
C11—C9—C10	113.5 (2)	H37A—C37—H37B	109.5
C33—C32—C31	122.7 (2)	H37A—C37—H37C	109.5
O3—C32—C31	118.5 (2)	H37B—C37—H37C	109.5
O3—C32—C33	118.7 (2)	O4—C39—H39A	109.5
C1—C10—C9	108.5 (2)	O4—C39—H39B	109.5
C5—C10—C9	109.4 (2)	O4—C39—H39C	109.5
C5—C10—C1	107.9 (2)	H39A—C39—H39B	109.5
C5—C10—C19	108.9 (2)	H39A—C39—H39C	109.5
C19—C10—C9	111.6 (2)	H39B—C39—H39C	109.5
C19—C10—C1	110.5 (2)	C25—C27—H27A	109.5
C20—C17—C13	118.6 (2)	C25—C27—H27B	109.5
C20—C17—C16	113.1 (2)	C25—C27—H27C	109.5
C20—C17—H17	107.2	H27A—C27—H27B	109.5
C13—C17—H17	107.2	H27A—C27—H27C	109.5
C16—C17—C13	102.8 (2)	H27B—C27—H27C	109.5
			
C(1)—C(2)—C(3)—O(1)	178.2 (2)	C(5)—C(10)—C(19)—H(19A)	69
C(1)—C(2)—C(3)—C(4)	57.2 (3)	C(5)—C(10)—C(19)—H(19B)	−51
C(1)—C(2)—C(3)—H(3)	−64	C(5)—C(10)—C(19)—H(19C)	−171
C(1)—C(10)—C(19)—H(19A)	−172	C(6)—C(5)—C(10)—C(1)	132.6 (3)
C(1)—C(10)—C(19)—H(19B)	68	C(6)—C(5)—C(10)—C(9)	14.8 (4)
C(1)—C(10)—C(19)—H(19C)	−52	C(6)—C(5)—C(10)—C(19)	−107.5 (3)
C(10)—C(1)—C(2)—C(3)	−56.7 (3)	C(6)—C(7)—C(8)—C(9)	−45.6 (3)
C(10)—C(5)—C(6)—C(7)	3.6 (5)	C(6)—C(7)—C(8)—C(14)	−167.4 (2)
C(10)—C(9)—C(11)—C(12)	173.4 (2)	C(6)—C(7)—C(8)—H(8)	73
C(10)—C(1)—C(2)—H(2A)	64	C(7)—C(8)—C(9)—C(10)	65.3 (3)
C(10)—C(1)—C(2)—H(2B)	−177	C(7)—C(8)—C(9)—C(11)	−166.0 (2)
C(10)—C(5)—C(6)—H(6)	−176	C(7)—C(8)—C(14)—C(13)	174.9 (2)
C(10)—C(9)—C(11)—H(11A)	−65	C(7)—C(8)—C(14)—C(15)	−62.9 (3)
C(10)—C(9)—C(11)—H(11B)	52	C(7)—C(8)—C(9)—H(9)	−50
C(11)—C(9)—C(10)—C(1)	65.2 (3)	C(7)—C(8)—C(14)—H(14)	57
C(11)—C(9)—C(10)—C(5)	−177.3 (2)	C(8)—C(9)—C(10)—C(1)	−166.0 (2)
C(11)—C(9)—C(10)—C(19)	−56.7 (3)	C(8)—C(9)—C(10)—C(5)	−48.6 (3)
C(11)—C(12)—C(13)—C(14)	59.5 (3)	C(8)—C(9)—C(10)—C(19)	72.1 (3)
C(11)—C(12)—C(13)—C(17)	170.1 (2)	C(8)—C(9)—C(11)—C(12)	46.3 (3)
C(11)—C(12)—C(13)—C(18)	−62.8 (3)	C(8)—C(14)—C(15)—C(16)	−159.4 (2)
C(12)—C(13)—C(14)—C(8)	−61.6 (3)	C(8)—C(9)—C(11)—H(11A)	168
C(12)—C(13)—C(14)—C(15)	169.6 (2)	C(8)—C(9)—C(11)—H(11B)	−75
C(12)—C(13)—C(17)—C(16)	−156.9 (2)	C(8)—C(14)—C(15)—H(15A)	81
C(12)—C(13)—C(17)—C(20)	77.4 (3)	C(8)—C(14)—C(15)—H(15B)	−40
C(12)—C(13)—C(14)—H(14)	57	C(9)—C(8)—C(14)—C(13)	54.4 (3)
C(12)—C(13)—C(17)—H(17)	−44	C(9)—C(8)—C(14)—C(15)	176.6 (2)
C(12)—C(13)—C(18)—H(18A)	64	C(9)—C(11)—C(12)—C(13)	−54.8 (3)
C(12)—C(13)—C(18)—H(18B)	−56	C(9)—C(8)—C(14)—H(14)	−64
C(12)—C(13)—C(18)—H(18C)	−176	C(9)—C(10)—C(19)—H(19A)	−52
C(13)—C(14)—C(15)—C(16)	−32.0 (3)	C(9)—C(10)—C(19)—H(19B)	−172
C(13)—C(17)—C(20)—C(21)	−65.2 (3)	C(9)—C(10)—C(19)—H(19C)	68
C(13)—C(17)—C(20)—C(22)	170.5 (2)	C(9)—C(11)—C(12)—H(12A)	66
C(13)—C(14)—C(15)—H(15A)	−151	C(9)—C(11)—C(12)—H(12B)	−176
C(13)—C(14)—C(15)—H(15B)	88	H(11A)—C(11)—C(12)—C(13)	−176
C(13)—C(17)—C(20)—H(20)	53	H(11A)—C(11)—C(12)—H(12A)	−55
C(14)—C(8)—C(9)—C(10)	−173.3 (2)	H(11A)—C(11)—C(12)—H(12B)	63
C(14)—C(8)—C(9)—C(11)	−44.6 (3)	H(11B)—C(11)—C(12)—C(13)	67
C(14)—C(13)—C(17)—C(16)	−42.6 (2)	H(11B)—C(11)—C(12)—H(12A)	−172
C(14)—C(13)—C(17)—C(20)	−168.3 (2)	H(11B)—C(11)—C(12)—H(12B)	−54
C(14)—C(15)—C(16)—C(17)	4.4 (3)	H(12A)—C(12)—C(13)—C(14)	−61
C(14)—C(8)—C(9)—H(9)	72	H(12A)—C(12)—C(13)—C(17)	49
C(14)—C(13)—C(17)—H(17)	70	H(12A)—C(12)—C(13)—C(18)	176
C(14)—C(13)—C(18)—H(18A)	−54	H(12B)—C(12)—C(13)—C(14)	−180
C(14)—C(13)—C(18)—H(18B)	−174	H(12B)—C(12)—C(13)—C(17)	−69
C(14)—C(13)—C(18)—H(18C)	66	H(12B)—C(12)—C(13)—C(18)	58
C(14)—C(15)—C(16)—H(16A)	124	H(14)—C(14)—C(15)—C(16)	81
C(14)—C(15)—C(16)—H(16B)	−116	H(14)—C(14)—C(15)—H(15A)	−38
C(15)—C(16)—C(17)—C(13)	24.1 (3)	H(14)—C(14)—C(15)—H(15B)	−159
C(15)—C(16)—C(17)—C(20)	153.3 (2)	H(15A)—C(15)—C(16)—C(17)	124
C(15)—C(16)—C(17)—H(17)	−89	H(15A)—C(15)—C(16)—H(16A)	−116
C(16)—C(17)—C(20)—C(21)	174.2 (2)	H(15A)—C(15)—C(16)—H(16B)	4
C(16)—C(17)—C(20)—C(22)	49.9 (3)	H(15B)—C(15)—C(16)—C(17)	−115
C(16)—C(17)—C(20)—H(20)	−68	H(15B)—C(15)—C(16)—H(16A)	5
C(17)—C(13)—C(14)—C(8)	175.3 (2)	H(15B)—C(15)—C(16)—H(16B)	125
C(17)—C(13)—C(14)—C(15)	46.5 (3)	H(16A)—C(16)—C(17)—C(13)	−96
C(17)—C(20)—C(22)—C(23)	−166.9 (2)	H(16A)—C(16)—C(17)—C(20)	33
C(17)—C(13)—C(14)—H(14)	−66	H(16A)—C(16)—C(17)—H(17)	151
C(17)—C(13)—C(18)—H(18A)	−164	H(16B)—C(16)—C(17)—C(13)	144
C(17)—C(13)—C(18)—H(18B)	76	H(16B)—C(16)—C(17)—C(20)	−87
C(17)—C(13)—C(18)—H(18C)	−44	H(16B)—C(16)—C(17)—H(17)	31
C(17)—C(20)—C(21)—H(21A)	−48	H(17)—C(17)—C(20)—C(21)	56
C(17)—C(20)—C(21)—H(21B)	−168	H(17)—C(17)—C(20)—C(22)	−68
C(17)—C(20)—C(21)—H(21C)	72	H(17)—C(17)—C(20)—H(20)	174
C(17)—C(20)—C(22)—H(22A)	−45	H(1A)—C(1)—C(2)—C(3)	−178
C(17)—C(20)—C(22)—H(22B)	72	H(1A)—C(1)—C(2)—H(2A)	−58
C(18)—C(13)—C(14)—C(8)	59.5 (3)	H(1A)—C(1)—C(2)—H(2B)	61
C(18)—C(13)—C(14)—C(15)	−69.3 (3)	H(1A)—C(1)—C(10)—C(5)	172
C(18)—C(13)—C(17)—C(16)	75.4 (2)	H(1A)—C(1)—C(10)—C(9)	−69
C(18)—C(13)—C(17)—C(20)	−50.3 (3)	H(1A)—C(1)—C(10)—C(19)	53
C(18)—C(13)—C(14)—H(14)	178	H(1B)—C(1)—C(2)—C(3)	65
C(18)—C(13)—C(17)—H(17)	−172	H(1B)—C(1)—C(2)—H(2A)	−174
C(2)—C(1)—C(10)—C(5)	50.6 (3)	H(1B)—C(1)—C(2)—H(2B)	−56
C(2)—C(1)—C(10)—C(9)	169.0 (2)	H(1B)—C(1)—C(10)—C(5)	−71
C(2)—C(1)—C(10)—C(19)	−68.4 (3)	H(1B)—C(1)—C(10)—C(9)	47
C(2)—C(3)—C(4)—C(5)	−55.3 (3)	H(1B)—C(1)—C(10)—C(19)	170
C(2)—C(3)—C(4)—H(4A)	−176	H(20)—C(20)—C(21)—H(21A)	−166
C(2)—C(3)—C(4)—H(4B)	66	H(20)—C(20)—C(21)—H(21B)	74
C(20)—C(22)—C(23)—C(24)	172.8 (3)	H(20)—C(20)—C(21)—H(21C)	−46
C(20)—C(22)—C(23)—H(23A)	−66	H(20)—C(20)—C(22)—C(23)	−49
C(20)—C(22)—C(23)—H(23B)	51	H(20)—C(20)—C(22)—H(22A)	73
C(21)—C(20)—C(22)—C(23)	68.1 (3)	H(20)—C(20)—C(22)—H(22B)	−170
C(21)—C(20)—C(22)—H(22A)	−170	H(22A)—C(22)—C(23)—C(24)	51
C(21)—C(20)—C(22)—H(22B)	−53	H(22A)—C(22)—C(23)—H(23A)	173
C(22)—C(23)—C(24)—C(25)	−175.9 (3)	H(22A)—C(22)—C(23)—H(23B)	−70
C(22)—C(20)—C(21)—H(21A)	77	H(22B)—C(22)—C(23)—C(24)	−66
C(22)—C(20)—C(21)—H(21B)	−43	H(22B)—C(22)—C(23)—H(23A)	56
C(22)—C(20)—C(21)—H(21C)	−163	H(22B)—C(22)—C(23)—H(23B)	173
C(22)—C(23)—C(24)—H(24A)	62	H(23A)—C(23)—C(24)—C(25)	63
C(22)—C(23)—C(24)—H(24B)	−54	H(23A)—C(23)—C(24)—H(24A)	−59
C(23)—C(24)—C(25)—C(26)	66.4 (4)	H(23A)—C(23)—C(24)—H(24B)	−176
C(23)—C(24)—C(25)—C(27)	−168.4 (3)	H(23B)—C(23)—C(24)—C(25)	−54
C(23)—C(24)—C(25)—H(25)	−51	H(23B)—C(23)—C(24)—H(24A)	−176
C(24)—C(25)—C(26)—H(26A)	−58	H(23B)—C(23)—C(24)—H(24B)	67
C(24)—C(25)—C(26)—H(26B)	−178	H(24A)—C(24)—C(25)—C(26)	−172
C(24)—C(25)—C(26)—H(26C)	62	H(24A)—C(24)—C(25)—C(27)	−47
C(24)—C(25)—C(27)—H(27A)	−67	H(24A)—C(24)—C(25)—H(25)	70
C(24)—C(25)—C(27)—H(27B)	173	H(24B)—C(24)—C(25)—C(26)	−55
C(24)—C(25)—C(27)—H(27C)	53	H(24B)—C(24)—C(25)—C(27)	70
C(26)—C(25)—C(27)—H(27A)	59	H(24B)—C(24)—C(25)—H(25)	−173
C(26)—C(25)—C(27)—H(27B)	−61	H(25)—C(25)—C(26)—H(26A)	60
C(26)—C(25)—C(27)—H(27C)	179	H(25)—C(25)—C(26)—H(26B)	−60
C(27)—C(25)—C(26)—H(26A)	177	H(25)—C(25)—C(26)—H(26C)	180
C(27)—C(25)—C(26)—H(26B)	57	H(25)—C(25)—C(27)—H(27A)	176
C(27)—C(25)—C(26)—H(26C)	−63	H(25)—C(25)—C(27)—H(27B)	56
C(28)—O(1)—C(3)—C(2)	164.1 (2)	H(25)—C(25)—C(27)—H(27C)	−64
C(28)—O(1)—C(3)—C(4)	−75.2 (3)	H(29)—C(29)—C(30)—C(31)	1
C(28)—C(29)—C(30)—C(31)	−179.1 (3)	H(29)—C(29)—C(30)—H(30)	−179
C(28)—O(1)—C(3)—H(3)	46	H(2A)—C(2)—C(3)—O(1)	57
C(28)—C(29)—C(30)—H(30)	1	H(2A)—C(2)—C(3)—C(4)	−63
C(29)—C(30)—C(31)—C(32)	−178.0 (3)	H(2A)—C(2)—C(3)—H(3)	176
C(29)—C(30)—C(31)—C(36)	1.7 (5)	H(2B)—C(2)—C(3)—O(1)	−61
C(3)—O(1)—C(28)—O(2)	1.3 (4)	H(2B)—C(2)—C(3)—C(4)	178
C(3)—O(1)—C(28)—C(29)	−177.3 (2)	H(2B)—C(2)—C(3)—H(3)	57
C(3)—C(4)—C(5)—C(6)	−128.5 (3)	H(3)—C(3)—C(4)—C(5)	66
C(3)—C(4)—C(5)—C(10)	52.8 (3)	H(3)—C(3)—C(4)—H(4A)	−55
C(30)—C(31)—C(32)—O(3)	2.3 (4)	H(3)—C(3)—C(4)—H(4B)	−173
C(30)—C(31)—C(32)—C(33)	178.7 (3)	H(30)—C(30)—C(31)—C(32)	2
C(30)—C(31)—C(36)—C(35)	−179.9 (3)	H(30)—C(30)—C(31)—C(36)	−178
C(30)—C(31)—C(36)—H(36)	0	H(35)—C(35)—C(36)—C(31)	−179
C(31)—C(32)—C(33)—C(34)	1.9 (4)	H(35)—C(35)—C(36)—H(36)	1
C(31)—C(32)—C(33)—C(38)	−174.4 (3)	H(4A)—C(4)—C(5)—C(6)	−7
C(32)—C(31)—C(36)—C(35)	−0.2 (4)	H(4A)—C(4)—C(5)—C(10)	174
C(32)—C(33)—C(34)—O(4)	179.8 (2)	H(4B)—C(4)—C(5)—C(6)	111
C(32)—C(33)—C(34)—C(35)	−1.5 (4)	H(4B)—C(4)—C(5)—C(10)	−68
C(32)—O(3)—C(37)—H(37A)	61	H(6)—C(6)—C(7)—C(8)	−168
C(32)—O(3)—C(37)—H(37B)	−59	H(6)—C(6)—C(7)—H(7A)	71
C(32)—O(3)—C(37)—H(37C)	−179	H(6)—C(6)—C(7)—H(7B)	−47
C(32)—C(31)—C(36)—H(36)	180	H(7A)—C(7)—C(8)—C(9)	76
C(32)—C(33)—C(38)—H(38A)	134	H(7A)—C(7)—C(8)—C(14)	−46
C(32)—C(33)—C(38)—H(38B)	14	H(7A)—C(7)—C(8)—H(8)	−166
C(32)—C(33)—C(38)—H(38C)	−106	H(7B)—C(7)—C(8)—C(9)	−167
C(33)—C(34)—C(35)—C(36)	0.3 (4)	H(7B)—C(7)—C(8)—C(14)	72
C(33)—C(34)—C(35)—H(35)	−180	H(7B)—C(7)—C(8)—H(8)	−48
C(34)—C(35)—C(36)—C(31)	0.6 (4)	H(8)—C(8)—C(9)—C(10)	−53
C(34)—O(4)—C(39)—H(39A)	−55	H(8)—C(8)—C(9)—C(11)	75
C(34)—O(4)—C(39)—H(39B)	−175	H(8)—C(8)—C(9)—H(9)	−168
C(34)—O(4)—C(39)—H(39C)	65	H(8)—C(8)—C(14)—C(13)	−66
C(34)—C(33)—C(38)—H(38A)	−42	H(8)—C(8)—C(14)—C(15)	57
C(34)—C(33)—C(38)—H(38B)	−162	H(8)—C(8)—C(14)—H(14)	176
C(34)—C(33)—C(38)—H(38C)	78	H(9)—C(9)—C(10)—C(1)	−51
C(34)—C(35)—C(36)—H(36)	−179	H(9)—C(9)—C(10)—C(5)	66
C(36)—C(31)—C(32)—O(3)	−177.4 (2)	H(9)—C(9)—C(10)—C(19)	−173
C(36)—C(31)—C(32)—C(33)	−1.1 (4)	H(9)—C(9)—C(11)—C(12)	−70
C(37)—O(3)—C(32)—C(31)	−103.0 (3)	H(9)—C(9)—C(11)—H(11A)	51
C(37)—O(3)—C(32)—C(33)	80.5 (3)	H(9)—C(9)—C(11)—H(11B)	168
C(38)—C(33)—C(34)—O(4)	−3.9 (4)	O(1)—C(3)—C(4)—C(5)	−173.1 (2)
C(38)—C(33)—C(34)—C(35)	174.9 (3)	O(1)—C(28)—C(29)—C(30)	−179.8 (3)
C(39)—O(4)—C(34)—C(33)	174.4 (3)	O(1)—C(3)—C(4)—H(4A)	66
C(39)—O(4)—C(34)—C(35)	−4.4 (4)	O(1)—C(3)—C(4)—H(4B)	−52
C(4)—C(5)—C(6)—C(7)	−175.1 (3)	O(1)—C(28)—C(29)—H(29)	0
C(4)—C(5)—C(10)—C(1)	−48.7 (3)	O(2)—C(28)—C(29)—C(30)	1.7 (5)
C(4)—C(5)—C(10)—C(9)	−166.5 (2)	O(2)—C(28)—C(29)—H(29)	−178
C(4)—C(5)—C(10)—C(19)	71.2 (3)	O(3)—C(32)—C(33)—C(34)	178.3 (2)
C(4)—C(5)—C(6)—H(6)	5	O(3)—C(32)—C(33)—C(38)	2.0 (4)
C(5)—C(6)—C(7)—C(8)	12.4 (4)	O(4)—C(34)—C(35)—C(36)	178.9 (3)
C(5)—C(6)—C(7)—H(7A)	−109	O(4)—C(34)—C(35)—H(35)	−1
C(5)—C(6)—C(7)—H(7B)	133		

### Hydrogen-bond geometry (Å, º)

Cg1 is the centroid of the C31–C36 ring.

**Table:**

*D*—H···*A*	*D*—H	H···*A*	*D*···*A*	*D*—H···*A*
C4—H4*A*···O2	0.99	2.58	3.104 (3)	113
C30—H30···O2	0.95	2.56	2.881 (3)	100
C30—H30···O3	0.95	2.45	2.814 (3)	103
C38—H38*B*···O3	0.98	2.44	2.870 (3)	106
C21—H21*A*···O3^i^	0.98	2.57	3.399 (3)	143
C36—H36···O3^ii^	0.95	2.51	3.431 (3)	164
C22—H22*B*···*Cg*1^i^	0.99	2.77	3.756 (3)	173

Symmetry codes: (i) *x*−1/2, −*y*+3/2, −*z*+1; (ii) −*x*+1, *y*+1/2, −*z*+3/2.
