# Supplementary figures and images for: Crystal structure of cholest-5-en-3β-yl 3-(2,4-dimeth­oxy-3-methyl­phen­yl)prop-2-enoate
Source: Acta Crystallogr E Crystallogr Commun. 2015 Jan 10;71(Pt 2):o92–3. doi: 10.1107/S2056989014028278 (PMC4384600; doi:10.1107/S2056989014028278)

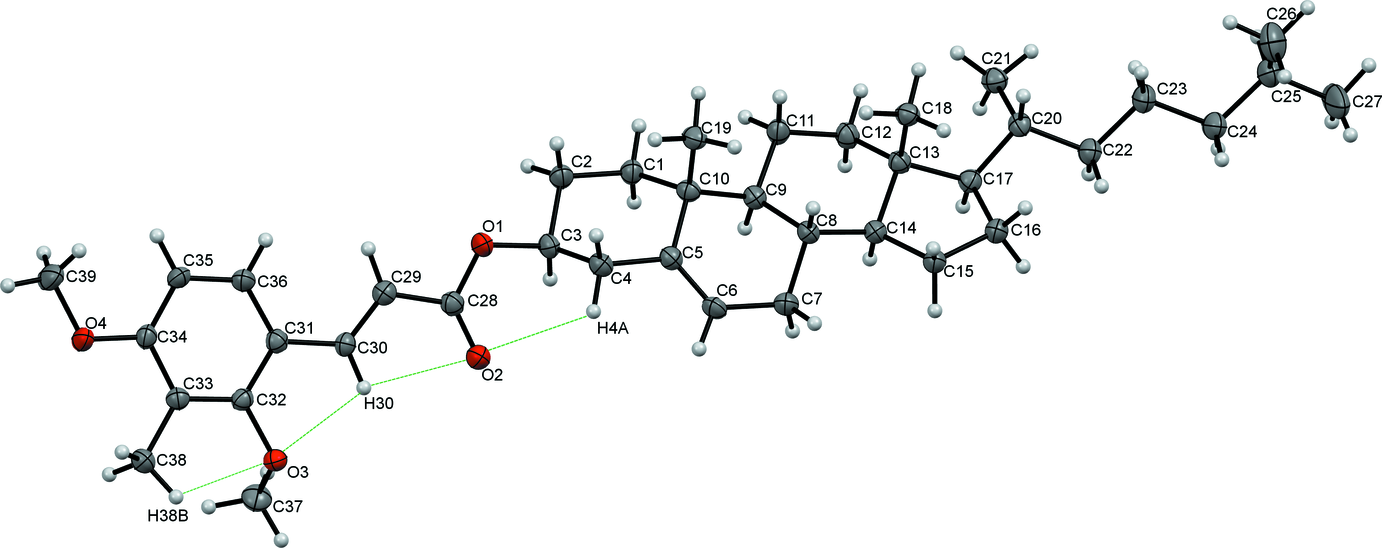

Supplement: Supplementary file 3 [file e-71-00o92-fig1.tif]

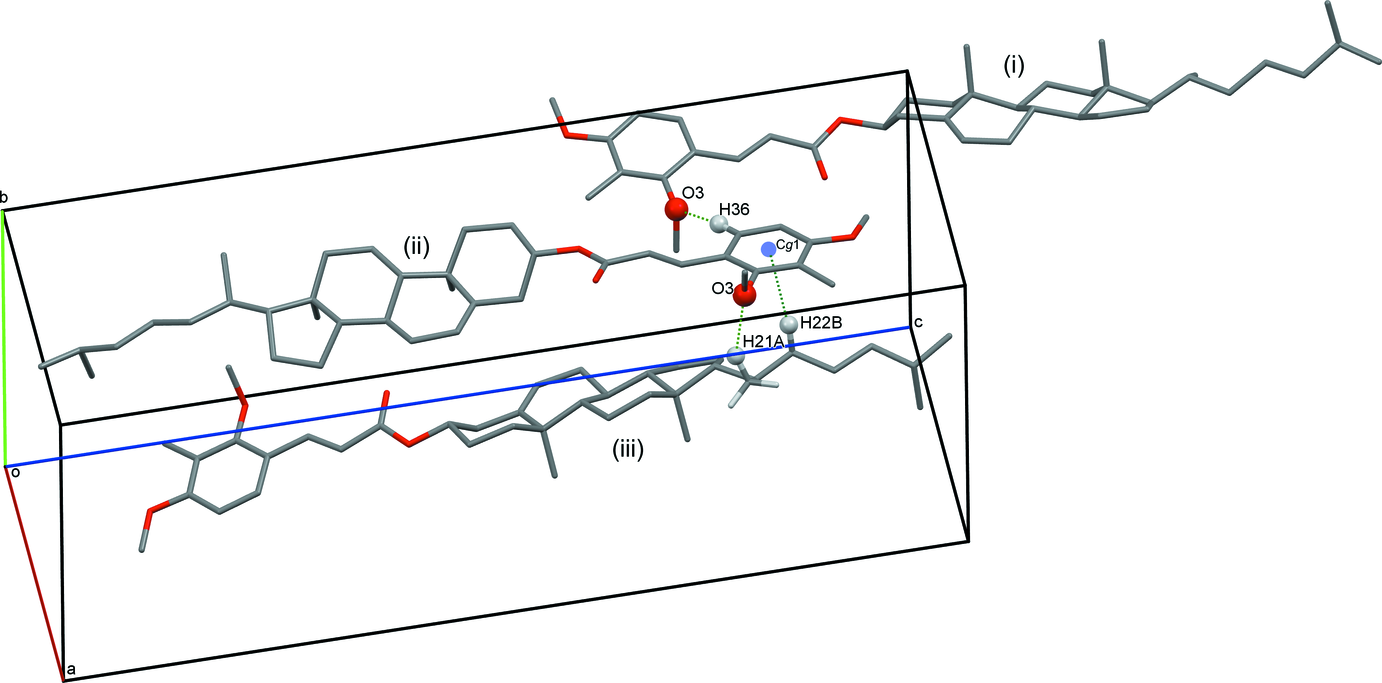

Supplement: Supplementary file 4 [file e-71-00o92-fig2.tif]

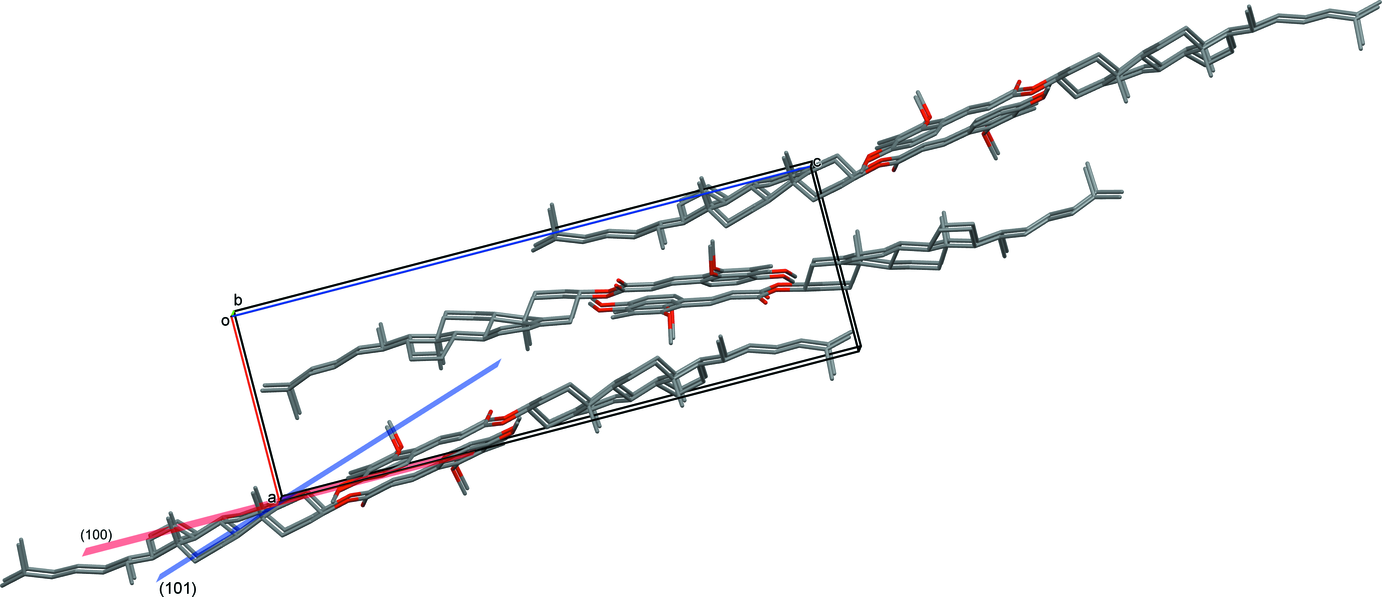

Supplement: Supplementary file 5 [file e-71-00o92-fig3.tif]
